# Biopolymers as a Potential Alternative for the Retention of Pollutants from Vinasse: An In Silico Approach

**DOI:** 10.3390/polym16010011

**Published:** 2023-12-19

**Authors:** Yesid Aristizabal, Yhors Ciro, Yamil Liscano, Constain H. Salamanca, Jose Oñate-Garzón

**Affiliations:** 1 Grupo de Investigación en Química y Biotecnología (QUIBIO), Facultad de Ciencias Básicas, Universidad Santiago de Cali, Cali 760035, Colombia; yesid.aristizabal00@usc.edu.co (Y.A.); yhors.ciro00@usc.edu.co (Y.C.); 2 Grupo de Investigación en Salud Integral (GISI), Departamento Facultad de Salud, Universidad Santiago de Cali, Cali 760035, Colombia; yamil.liscano00@usc.edu.co; 3 Grupo de Investigación Ciencia de Materiales Avanzados, Escuela de Química, Facultad de Ciencias, Universidad Nacional de Colombia sede Medellín, Medellín 050034, Colombia; 4 Grupo de Investigación Biopolimer, Departamento de Farmacia, Facultas de Ciencias Farmacéuticas y Alimentarias, Universidad de Antioquia, Calle 67 #53-108, Medellín 050034, Colombia

**Keywords:** vinasse, biopolymers, pollutant flocculation, molecular dynamics

## Abstract

Vinasse, a waste from the bioethanol industry, presents a crucial environmental challenge due to its high organic matter content, which is difficult to biodegrade. Currently, no sustainable alternatives are available for treating the amount of vinasse generated. Conversely, biopolymers such as cellulose, carboxymethylcellulose, and chitosan are emerging as an interesting alternative for vinasse control due to their flocculating capacity against several organic compounds. This study seeks to determine the thermodynamic behavior of in silico interactions among three biopolymers (cellulose, carboxymethylcellulose, and chitosan) regarding 15 organic compounds found in vinasse. For this, the Particle Mesh Ewald (PME) method was used in association with the Verlet cutoff scheme, wherein the Gibbs free energy (ΔG) was calculated over a 50 ns simulation period. The findings revealed that cellulose showed a strong affinity for flavonoids like cyanidin, with a maximum free energy of −84 kJ/mol and a minimum of −55 kJ/mol observed with phenolic acids and other flavonoids. In contrast, chitosan displayed the highest interactions with phenolic acids, such as gallic acid, reaching −590 kJ/mol. However, with 3-methoxy-4-hydroxyphenyl glycol (MHPG), it reached an energy of −70 kJ/mol. The interaction energy for flavonoid ranged from −105 to −96 kJ/mol. Finally, carboxymethylcellulose (CMC) demonstrated an interaction energy with isoquercetin of −238 kJ/mol, while interactions with other flavonoids were almost negligible. Alternatively, CMC exhibited an interaction energy of −124 kJ/mol with MHPG, while it was less favorable with other phenolic acids with minimal interactions. These results suggest that there are favorable interactions for the interfacial sorption of vinasse contaminants onto biopolymers, indicating their potential for use in the removal of contaminants from the effluents of the bioethanol industry.

## 1. Introduction

The increasing demand for biofuels like ethanol has presented a significant global environmental challenge. Reports indicate that approximately 13 L of waste, known as “vinasse”, are generated for every liter of bioethanol produced [1]. Vinasse is composed of a high content of organic matter, where phenolic acids and polyphenolic flavonoids are its main components [2]. These compounds have usually been described as having antioxidant and antimicrobial biological activity. However, when these compounds are in large quantities, they become phytotoxic, limiting their own biodegradation process [3]. Managing such organic waste has become a complex challenge due to its inherent difficulty in biodegrading.

Furthermore, studies show that when vinasse is discharged near or directly into water sources, it causes adverse environmental consequences due to its high chemical and biological oxygen demand, which affects the physicochemical and microbiological characteristics of water bodies [4].

Although several alternatives have been proposed for vinasse bioremediation, their implementation has proven ineffective due to the substantial volumes of waste generated, leading to cost-intensive and inefficient processes [5]. Therefore, we must continue searching for novel and enhanced solutions for handling this bioindustrial waste, prioritizing characteristics such as low environmental impact, high sustainability, and a balanced cost-effectiveness ratio as the cornerstones of ongoing research. Within this context, biopolymers emerge as a promising alternative. They primarily comprise nontoxic, biodegradable compounds with sustainable and renewable characteristics, offering cost-effective materials with remarkable potential for capturing organic contaminants [6,7]. It is important to highlight that although there are currently several cost-effective alternatives in the treatment of vinasse, such as clays or aluminum sulfate, the projection of the use of biopolymers as potential decontamination systems is based mainly on their origin source. In this way, polymeric materials such as cellulose and chitin can be obtained from agroindustrial biowaste, which can subsequently be transformed into other types of biopolymers such as modified cellulose and chitosan. In the specific case of Colombia, which is a country widely known as a producer of coffee and, to a lesser extent, sugar, rice, cotton, corn, and shrimp, the waste generated in these agroindustries could be used to obtain this type of polymeric material. For example, in the case of coffee, this industry only uses the grain, which is equivalent to 5% of the fruit, leaving 95% waste, where approximately 60% of this is cellulose [8,9].

Cellulose has been widely studied, and it has been reported that, in its natural state, it can retain various organic substances [10,11], i.e., aromatic compounds such as polyphenols that are present in vinasse [12]. On the other hand, cellulose can be easily structurally modified to enhance several physicochemical and functional properties. One of these modified cellulose biopolymers is carboxymethylcellulose (CMC), which is a water-soluble anionic polyelectrolyte widely recognized for its excellent flocculating capacity on various industrial contaminants, primarily of cationic nature [13]. In contrast, chitosan also belongs to the polysaccharide-type biopolymer family and is primarily extracted from the exoskeleton of crustaceans through the alkaline deacetylation of chitin [14]. It is the second-most abundant natural organic source on Earth after cellulose biopolymers [15]. In Colombia, aquaculture produces an average of 5000 tons of shrimp per year between 2012 and 2020, of which between 48% and 60% are discarded, which generates public health and environmental problems due to their decomposition and the attraction of vectors transmitting infectious diseases [16]. However, among this waste, the exoskeletons represent between 30% and 48% of the weight [17], which are raw materials used to produce chitin and chitosan. This modified biopolymer is a cationic polyelectrolyte with diverse applications, notably its effectiveness as a flocculating agent in wastewater treatment and sludge dewatering [18].

Thus, the use of biopolymers obtained from industrial waste can be projected as an alternative system for the treatment itself or of other agroindustrial waste such as vinasse. Therefore, waste from coffee that has a high cellulose content could be extracted to be used directly or could be transformed into cellulose derivatives such as carboxymethyl cellulose-CMC. While waste from shrimp exoskeletons can be transformed into chitin and chitosan polymers.

**Figure 1:**
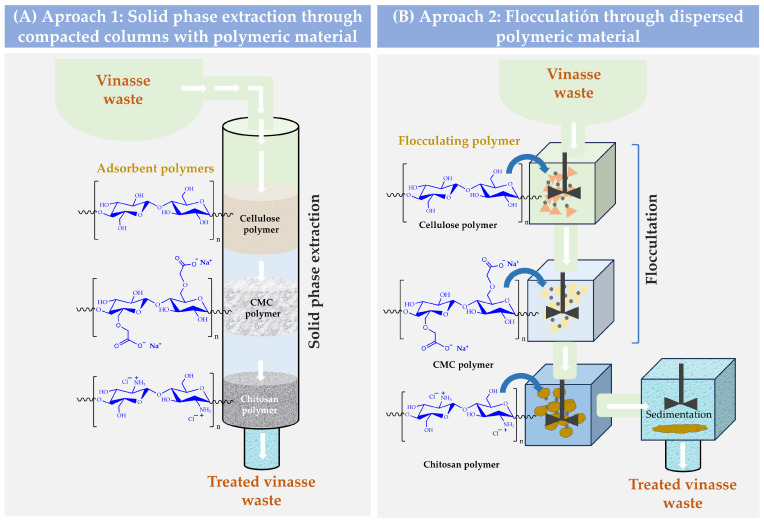
Schematization of possible ways for the treatment of vinasse waste through polymeric interfacial adsorption phenomena. (**A**) Purification through solid-phase extraction. (**B**) Purification by flocculation sedimentation.

In this way, it is possible to think of a circular waste treatment model where agroindustrial waste could be used to treat other agroindustrial waste. Thus, cellulose and its derivatives can be obtained from coffee waste, while chitosan can be obtained from shrimp exoskeleton waste. In this way, these polymers could be used under two approaches for the treatment of vinasse. For example, they could be used as compacted materials inside a column where the vinasse is passed as a fluid, or they could be used as flocculating-coagulant agents, which are directly added to the vinasse (Figure 1). In both cases, the purification principle would be based on the polymer retention capacity of the different organic molecules of the vinasse.

**Figure 2:**
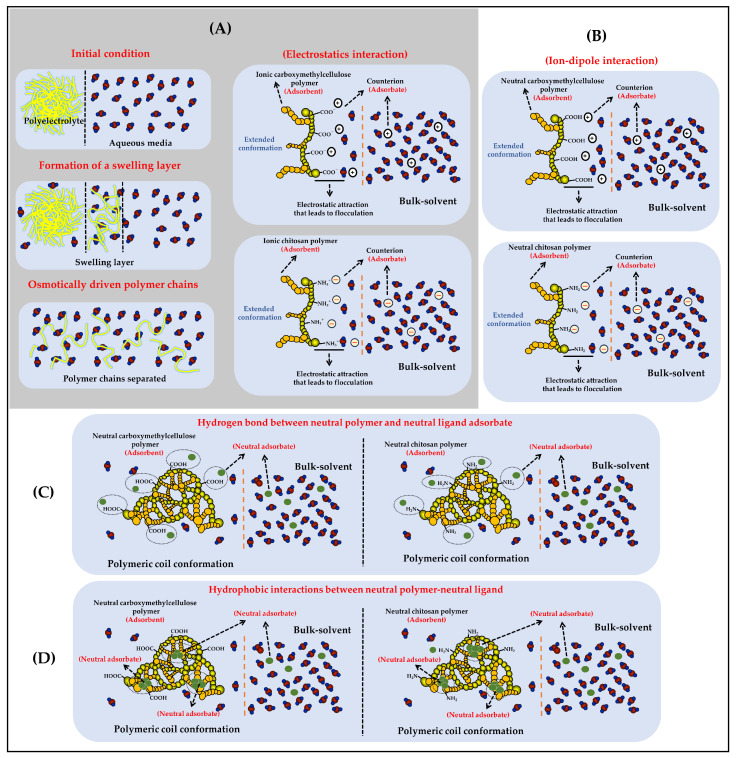
Schematization of the different types of interactions that can take place between polymers derived from cellulose (cellulose and carboxymethyl cellulose) and chitosan with various components of the vinasse. (**A**) Phenomenon of separation of polymer chains and formation of electrostatic interactions between polyelectrolytes and ionic sorbent ligands. (**B**) Formation of ion-dipole interactions between neutral polymers and ionic sorbent ligands. (**C**) Formation of neutral hydrogen bonding interactions between polar neutral polymers and polar neural sorbent ligands. (**D**) Formation of hydrophobic interactions between nonpolar polymer region and nonpolar sorbent ligands.

In relation to the solid phase extraction process, this depends on several factors, such as the characteristics of the fluid (concentration, viscosity, flow rate, i.a.) [19], as well as the characteristics of the polymeric material (size, degree of cross-linking, surface and granulometric properties, i.a.) [20]. For example, polymeric materials with a high molecular weight as well as with a high degree of cross-linking tend to remain in a solid state, leading to difficult disintegration and dispersion in liquid media [21]. Likewise, polymeric materials with ease of compaction, high porosity, and various functional groups can act with adsorption, extraction, and preconcentration systems [22]. In contrast, polymeric materials consisting of individual chains with medium and low molecular weights, as well as with polar functional groups (OH, COOH, NH2, i.a.), can interact favorably with the aqueous medium, going through a process of swelling and separation of their individual chains to end in a heterogeneous (colloidal suspension) or homogeneous (polymeric solubilization) dispersion (Figure 2). Regarding flocculating capacity, it relates to a colloidal physicochemical property involving an interfacial interaction phenomenon in an aqueous medium between a porous or high-surface-area material (sorbent) and a target compound intended to be retained or extracted (sorbate) from the aqueous phase [23]. This flocculation phenomenon can occur in different ways depending on the described characteristics and both the sorbent and the sorbate [23,24]. For instance, the most common and effective form of flocculation happens when the material serving as the sorbent, typically an ionic polymer (polyelectrolyte), interacts with a sorbate that is also ionic but carries an opposite charge (electrostatic interactions). In this specific scenario, the initial step involves the complete dissolution of the polymeric material in the aqueous medium. This dissolution process comprises several substages, including polymer swelling, the disintegration or separation of polymer chains, the dissociation of ionizable groups, and the elongation of the main polymer chains due to ionization. Subsequently, each separated and elongated polymer chain creates a substantial interfacial area between the polymer and aqueous medium, where the sorption phenomenon then occurs. Similarly, the sorbate must also be previously dissolved and ionized, carrying an opposite charge to that of the sorbing polyelectrolyte, to establish a potent long-range electrostatic interaction effect. As a result, when the polymeric sorbent and the ionized sorbate reach attractive interaction distances, the electrostatic complexation process occurs with high intensity. This interaction displaces the water molecules that were previously solvating the sorbing polyelectrolyte and the sorbate, leading to a dramatic decrease in the solubility of the polyelectrolyte-sorbate complex. This results in desolvation and precipitation, which can subsequently be separated through decantation and filtration processes (Figure 2A).

In fact, apart from electrostatic interactions, there can also be other types of less potent intermolecular interactions between the sorbent polymer and the sorbate. These include ion-dipole interactions, hydrogen bonding interactions, and London dispersion interactions, here called hydrophobic interactions.

Ion-dipole interactions occur between an ion from a functional group of a potential electrolyte (weak acids and bases) and a permanent dipole from a neutral functional group. In addition, the dipole from the neutral group must have an opposite character to the charge described by the ionized group to establish an attractive interaction between the sorbent and the sorbate. Moreover, the efficiency of flocculation is greatly influenced by which component serves as the ion and which as the dipole. For example, when the sorbent is a nonionic or neutral polymer, it tends to adopt compact or coiled conformations in the aqueous medium, resulting in a smaller interfacial area for sorbent and sorbate interaction and, consequently, reduced flocculation efficiency. In contrast, when the sorbent is a polyelectrolyte, it can achieve a larger interfacial area due to the stretching of its polymer chains based on its degree of ionization and the pH of the medium. Consequently, it can interact more efficiently with a neutral sorbate containing dipoles of an opposite character to the charge described by the sorbent (Figure 2B).

Another form of interaction, much weaker than electrostatic and ion-dipole interactions, involves the formation of hydrogen bonds. In this scenario, both the sorbent and the sorbate must be dissolved in the aqueous medium and have a neutral character defined by their functional groups. Furthermore, these groups must have the capacity to establish multiple hydrogen bonds, which should in turn generate a greater level of interaction intensity than that provided by the aqueous medium, thus facilitating the formation of the sorbent–sorbate complex, leading to desolvation and precipitation (Figure 2C).

Finally, the interactions with the lowest intensity are those occurring between aliphatic and aromatic functional groups, giving rise to London dispersion forces or hydrophobic forces. This can occur when the sorbent polymer, as well as the sorbate, adopt conformations or configurations in which some of their aliphatic or aromatic groups are oriented toward the aqueous medium, leading to extremely weak interaction processes between the sorbate and the sorbent. Hydrophobic interaction forces, as well as those mediated by hydrogen bonding, typically exhibit very low flocculation efficiencies since the interaction intensity does not consistently lead to desolvation and subsequent precipitation, as previously described for electrostatic and ion-dipole interactions between the sorbent and the sorbate (Figure 2D).

As mentioned above, a practical way to assess the potential of various biopolymeric materials that can act as flocculating systems for vinasse is through the thermodynamic study of intermolecular interactions that can occur at the interfaces between the polymer and the aqueous medium, related to the sorption phenomenon of the ligand of interest. In this regard, the sorption phenomenon can be thermodynamically described by the Gibbs free energy (ΔG) generated during the interaction process between a sorbent (biopolymer) and a sorbate (ligand) [25]. As a result, molecular dynamics has become a dependable computational tool capable of providing detailed insights into the potential interactions between two systems acting as ligands over time. In this sense, interactions between polymers and contaminants have been assessed by molecular dynamics studies [26,27]. However, until now, there have been no in silico studies concerning potential interactions between vinasse contaminants and sorbent biopolymers intended as prospective contaminant retention agents. For this reason, this research study assessed the interaction effect of two sorbent materials derived from cellulose and chitosan with respect to 15 components present in vinasse. In this study, cellulose oligomers, the anionic polyelectrolyte carboxymethylcellulose, and the cationic polyelectrolyte chitosan were used as sorbents, while different types of phenols and phenolic acids were used as sorbates.

## 2. Materials and Methods

### 2.1. 3D Modeling of Sorbents

For the construction of each sorbent, the union of five monomeric units was used as the base to ensure the solubility of low-molecular-weight oligomers in an aqueous model. The cellulose sorbent was constructed using five units of D-glucose (PubChem CID: 5793), the chitosan sorbent using five units of D-Glucosamine (PubChem CID: 439213), and the carboxymethylcellulose (CMC) sorbent using five units of 6-O-carboxymethylglucose (PubChem CID: 87648953). To achieve the linear structure of the cellulose, chitosan, and carboxymethylcellulose sorbents, the monomeric units with 1,4-β glucosidic linkages were generated using the CHARMM-GUI platform in conjunction with the PDB Reader and Manipulator module [28].

### 2.2. Construction of the Sorbent–Sorbate Assembly Model

The Multicomponent Assembler module from the CHARMM-GUI web platform [29] was used to create a rectangular water box system with a volume of 1.25 × 10^5^ Å^3^ at a concentration of 0.30 M KCl and approximately 4000 water molecules (TIP3 molecules). The quantity of water molecules could decrease based on the molecular volume of each organic species within the system. A single sorbent oligomer and one molecule for each sorbate were added. With M representing the number of sorbates present in sugarcane vinasse and N representing the number of sorbents, a total of M × N simulated systems were obtained.

### 2.3. Molecular Dynamics Simulation

**Figure 3:**
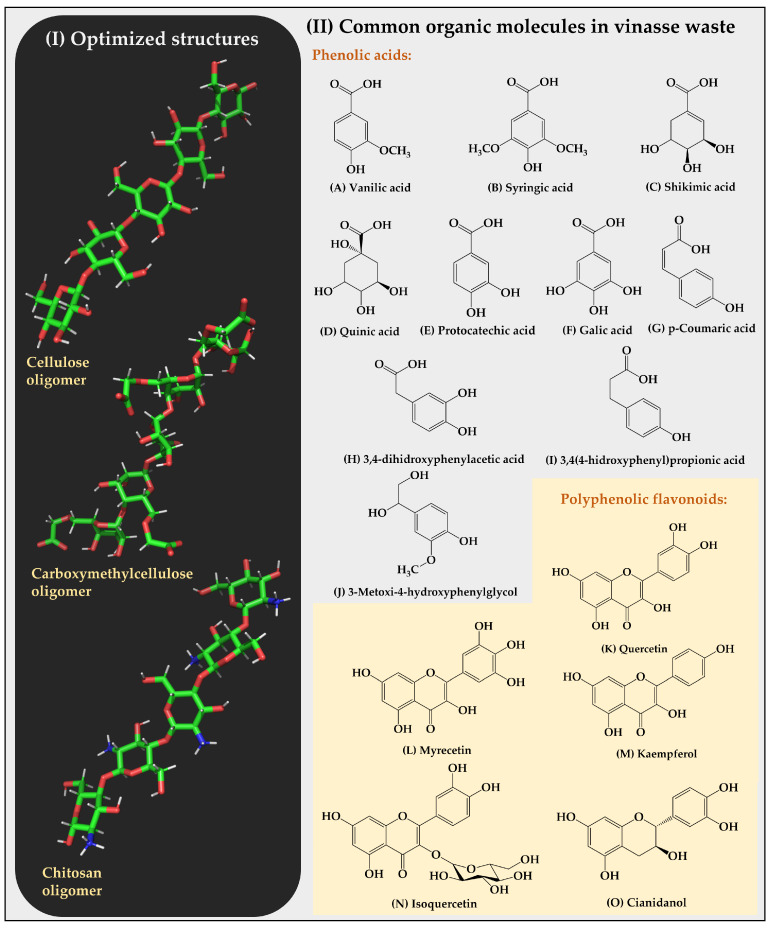
(**I**) Optimized structures of cellulose, carboxymethylcellulose, and chitosan oligomers. (**II**) Structures of the organic substrates of vinasse are classified into two categories, corresponding to phenolic acids and polyphenolic flavonoids.

The molecular dynamics simulations were conducted using GROMACS version 2022.2 [30]. The CHARMM36m force field was used at a temperature of 300 K. For the calculation of electrostatic potentials, the Particle Mesh Ewald (PME) method was used, and the Verlet cutoff scheme with the steepest descent algorithm that converges to a minimum energy value of 1000 kJ/mol was adopted [31,32]. The simulation was subdivided into three phases: (i) minimization, (ii) equilibration, and (iii) production. The minimization phase was conducted at a ratio of 5000 steps at 1 fs/step. The equilibration stage was conducted at a ratio of 125,000 steps at 1 fs/step, using an extended Nose–Hoover ensemble for temperature coupling in a canonical NVT-type ensemble [33,34]. The production stage was conducted at a ratio of 25,000,000 steps at 2 fs/step, using an extended Parrinello–Rahman ensemble for pressure coupling in a canonical NPT-type ensemble [35]. In subsequent analyses, the most frequent intermolecular interactions between the sorbents and sorbates were determined. These interactions were obtained in Discovery Studio, sampling data every 2 ns for relative frequency calculations [36]. The binding free energy was estimated using the Poisson–Boltzmann surface area (MM-PBSA) approach in molecular mechanics. For this purpose, the g_mmpbsa tool, which utilizes internal subroutines from the GROMACS package and APBS [37,38], was employed. The optimized structures of the cellulose, carboxymethylcellulose, and chitosan oligomers, as well as the structures of the organic substrates from the vinasse, are shown in Figure 3.

## 3. Results and Discussions

**Figure 4:**
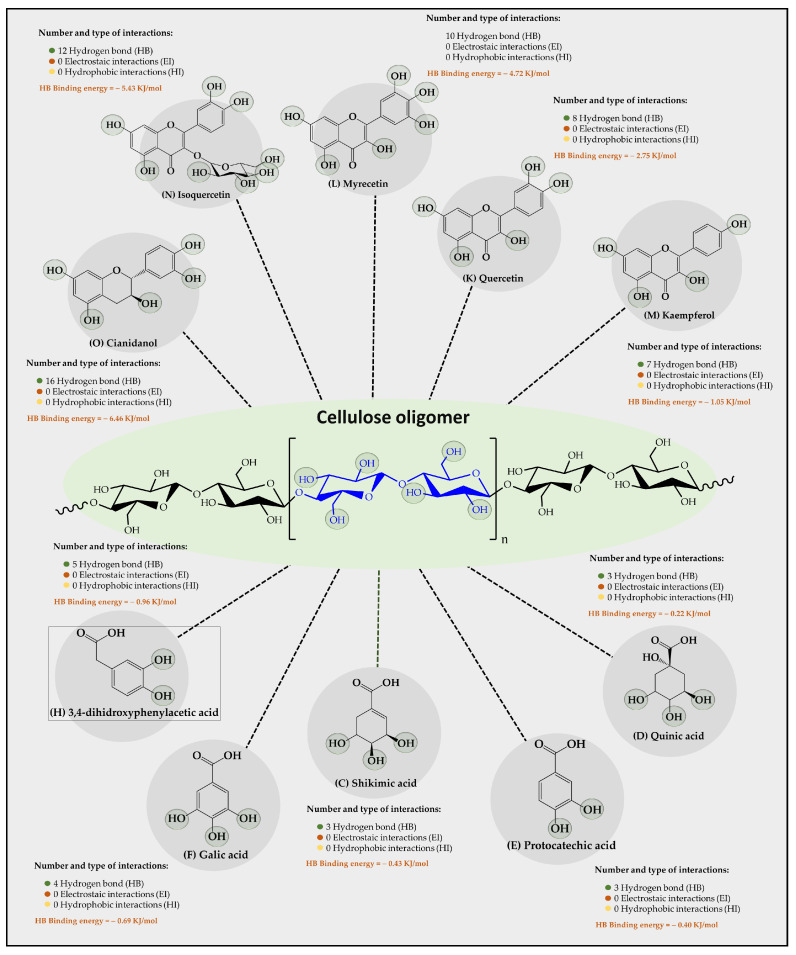
Results of the types of intermolecular interaction, as well as binding energies between cellulose oligomers and organic substrates from vinasse.

**Figure 5:**
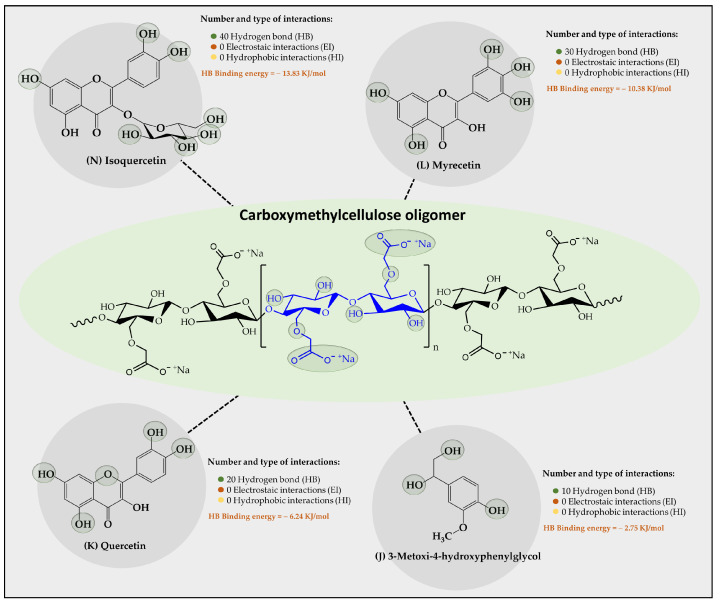
Results of the types of intermolecular interaction, as well as binding energies between the carboxymethylcellulose oligomers and the organic substrates from vinasse.

**Figure 6:**
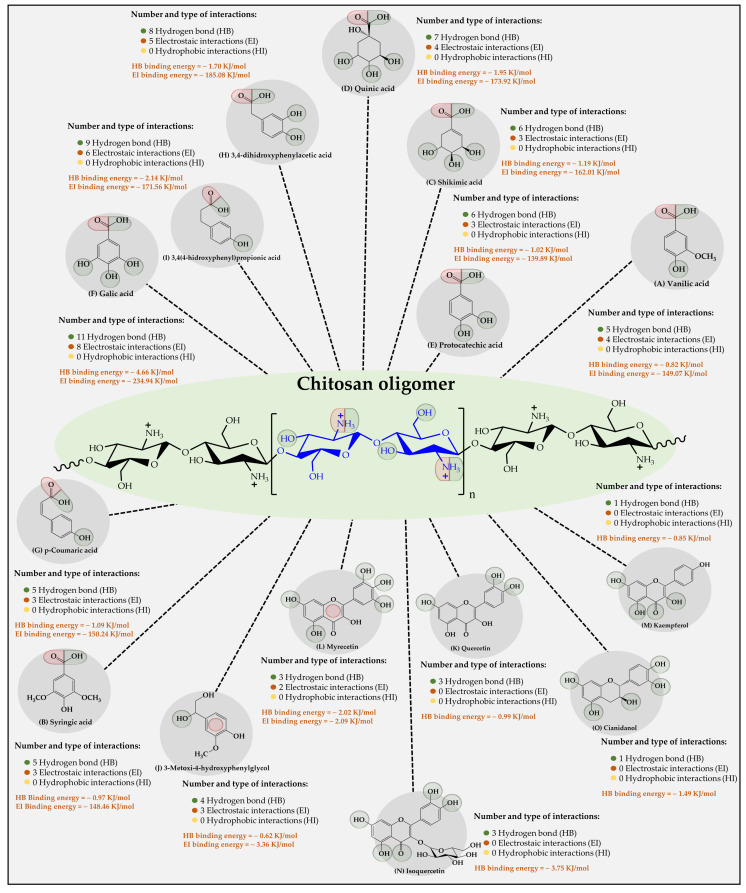
Results of the types of intermolecular interaction, as well as binding energies between the chitosan oligomers and the organic substrates from vinasse.

Results of an in silico study of the interaction of 15 organic substrates from vinasse with cellulose, carboxymethylcellulose, and chitosan oligomers are shown in Figure 4, Figure 5, and Figure 6, respectively. Results of the interaction between cellulose oligomers and organic substrates from vinasse (Figure 4) showed that it could only establish stable interactions over time with only 10 organic substrates. Furthermore, it was observed that the predominant type of interaction corresponded to hydrogen bonds (HBs) with relatively low binding energies. In this way, the polyphenolic flavonoids Cinadiol, Isoquertecin, Myricetin, Quercetin, and Kaempferol established 16, 12, 10, 8, and 7 HB interactions with binding energies of −6.46, −5.43, −4.72, −2.75, and −1.05 kJ/mol, respectively. In contrast, the phenolic acids 3,4-dihydroxyphenylacetic acid, gallic acid, shikimic acid, protocatechic acid, and quinic acid showed a lower number of interactions and binding energies corresponding to 5, 4, 3, 3, and 3 HB with binding energies values of −0.96, −0.69, −0.43, −0.40, and −0.22 kJ/mol, respectively. These results are very interesting because they suggest that cellulose oligomers have a greater affinity for polyphenolic flavonoids than for phenolic acids, which can be explained by considering the chemical composition and the solubility in the aqueous medium. In this way, both the oligomer and the organic substrates that are polyhydroxylated systems can form multiple HB interactions between themselves or with the aqueous medium, establishing a competitive interaction. Therefore, in the case of polyphenolic flavonoids that are characterized by having a larger size and low aqueous solubility [39], they tend to achieve higher binding energies with cellulose oligomers. In contrast, phenolic acids that are smaller in molecular size and have greater aqueous solubility [40] tend to remain longer in the aqueous medium, interacting slightly with the cellulose oligomer. Liu et al., [41] studied the adsorption of catechin onto cellulose, revealing that the adsorption capacities of cellulose for catechin were 2.70 and 2.82 mg/g at a pH of 2.0 and 7.0, respectively. Din et al. [10] removed organic matter from river water using natural cellulose from coconut fiber and palm oil fiber via adsorption isotherm and kinetic studies, reporting the adsorption capacities achieved, which were 15.67 and 30.8 mg/g, respectively, suggesting that it is a suitable substrate for the retention of organic contaminants in an aqueous medium.

Regarding the results of the interaction provided between the carboxymethylcellulose-CMC oligomer and the organic substrates from vinasse (Figure 5), a poor capability to establish interactions with most of the vinasse substrates was found, where only four organic substrates showed stable interactions over time. In this case, it was observed that the polyphenolic flavonoids Isoquertecin, Myricetin, and Quercetin, as well as the phenolic acid 3-mnetoxy-4hydroxyphenyl-glycol corresponded to those that described a moderate interaction, generating 40, 30, 20, and 10 HB interactions with binding energies of −13.83, −10.38, −6.24, and −275 kJ/mol, respectively. This limited capability for interaction between the oligomer and organic substrates can be explained considering that the CMC oligomer is an anionic polyelectrolyte with a high tendency towards aqueous solvation [42]. This characteristic leads to competition between oligomer-aqueous medium and oligomer-organic substrate interactions. Likewise, the degree of ionization of the oligomer also entails the formation of a larger electronic cloud, leading to an electrostatic repulsion effect that limits the approach of the organic substrates towards the oligomer. Furthermore, it was observed that the organic substrate-oligomer affinity is achieved mainly with polyphenolic flavonoids rather than with phenolic acids. This result can also be explained by analyzing the solubility of both compounds, where the phenolic acids have small sizes and high solubility, tending to remain more in the aqueous medium than in the oligomers. On the contrary, polyphenolic flavonoids have a large size and low solubility, tending to be poorly solvated, favoring the interaction with the oligomer. Evidence has been reported that suggests that CMC has the capacity to adsorb organic matter. For instance, Capanema et al. [43] prepared cross-linked carboxymethylcellulose hydrogels as adsorbents for the removal of organic dye pollutants, which exhibited adsorption efficiency above 90% (24 h) and a maximum removal capacity of methylene blue from 5 to 25 mg g^−1^ depending on the dye concentration.

The results of the interaction between the chitosan oligomer and the organic substrates from vinasse showed that it established stable interactions over time with each of the substrates (Figure 6). Furthermore, it was observed that two types of intermolecular oligomer-substrate binding interactions were formed, corresponding to (i) low-energy hydrogen bonds (HB) and (ii) high-energy ion-dipole and π-type electrostatic interactions (EI).

In this case, it was observed that, unlike cellulose and CMC oligomers, the substrate-ligand affinity is indifferent to the type of substrate. Additionally, it was found that the highest affinity was for the phenolic compound gallic acid (11 HB with a binding energy value of −4.66 kJ/mol and 8 EI with a binding energy value of −234.94 kJ/mol), followed by 3,4(4-hydroxyphenyl) propionic acid (9 HB with a binding energy value of −2.14 kJ/mol and 6 EI with a binding energy value of −171.56 kJ /mol), 3,4 dihydroxyphenylacetic acid (8 HB with a binding energy value of −1.70 kJ/mol and 5 EI with a binding energy value of −185.08 kJ/mol), quinic acid (7 HB with a binding energy value of −1.95 kJ/mol and 4 EI with a binding energy value of −173.92 kJ/mol), shikimic acid (6 HB with a binding energy value of −1.19 kJ/mol, and 3 EI with a binding energy value of −162.01 kJ/mol), protocatetechic acid (6 HB with a binding energy value of −1.02 kJ /mol and 3 EI with a binding energy value of −139.89 kJ/mol), vanilic acid (5 HB with a binding energy value of −0.82 kJ/mol and 4 EI with a binding energy value of −149.07 kJ/mol), p-Coumaric acid (5 HB with a binding energy value of −1.09 kJ/mol and 3 EI with a binding energy value of −150.24 kJ/mol), syringic acid (5 HB with a binding energy value of −0.97 kJ/mol and 3 EI with a binding energy value of −148.46 kJ/mol), 3-methoxy- 4-hydroxyphenylglycol (4 HB with a binding energy value of −0.62 kJ/mol and 3π-type EI with a binding energy value of −3.36 kJ/mol), Myrecetin (3 HB with a binding energy value of −2.02 kJ/mol and 2π-type EI with a binding energy value of −2.09 kJ/mol), Isoquercetin (3 HB with an energy value of binding of −3.75 kJ/mol), Quercetin (3 HB with a binding energy value of −0.99 kJ/mol), Cyanidanol (1 HB with a binding energy value of −1.49 kJ/mol), and Campeferol (1 HB with a binding energy value of −0.85 kJ/mol). These results suggest that the chitosan oligomer describes the greatest number of interactions with the organic substrates in vinasse, establishing several mechanisms of interaction, as well as different binding energies. Such results can be explained by virtue of the chemical nature of the oligomer, which has ammonium-type cations in its molecular structure, which allows it to establish different points of interaction with many of the negative dipoles formed in the organic compounds of the vinasse. These results are very similar to those found by Liudvinaviciute et al. [44], where the adsorption of rosmarinic acid (RA) from an aqueous solution on chitosan powder was explored. There, the adsorption of RA on chitosan powder occurred in two stages. In the first step of adsorption, the RA molecules are attached to the ionized amino groups on the surface of chitosan powder due to electrostatic interaction. In the next step, the adsorbed RA molecules become the sites for the adsorption of other RA molecules due to hydrophobic interaction. In another study, chitosan-macroalgae biocomposites as potential adsorbents of water-soluble hydrocarbons were used [45]. The authors reported that the removal capacities were 58.68, 16.64, and 6.13 mg g−1 for benzene, toluene, and naphthalene, respectively. Finally, it is important to highlight that none of the three oligomers established hydrophobic interactions (London dispersion forces) with organic substrates. This result can be explained considering that this type of interaction requires an extremely short interaction distance [46,47]. However, both oligomers and organic substrates consist of a wide variety of polar groups that generate complex electronic clouds that considerably limit the formation of such interactions.

## 4. Conclusions

Oligomers derived from biopolymers such as cellulose, carboxymethylcellulose, and chitosan displayed diverse interaction patterns regarding the chemical characteristics of the 15 compounds present in vinasse. In the case of the cellulose oligomer, it showed the capability to interact with a wide number of substrates from vinasses. Likewise, it was found that the predominant type of interaction between this oligomer and the organic substrates was hydrogen bonds with relatively low binding energies. Furthermore, it was found that the cellulose oligomer showed a slightly greater affinity for flavonoid-type organic substrates than for the phenolic acids of the vinasse. Regarding the carboxymethylcellulose oligomer, it showed a poor capability to interact stably with most substrates in vinasse. However, with the four substrates that established stable interactions, it was achieved through the formation of multiple hydrogen bonds. In the case of the chitosan oligomer, this showed a capability to interact with each of the substrates from the vinasses. Furthermore, this oligomer showed the capability to establish two types of oligomer-substrate interactions corresponding to low-energy hydrogen bonds and high-energy electrostatic interactions (ion-dipole and pi interactions). In this way, this study allows for the first approximation of the possible interaction behavior between polymeric materials such as cellulose, carboxymethylcellulose, and chitosan with various organic molecules that make up the vinasse waste, and therefore, project future experimental studies focused on evaluating the ability of such materials as sorption, extraction, and/or preconcentration systems for vinasse waste.

## Data Availability

Data are available in the manuscript.

## References

[1] Nadaleti W.C., Lourenço V.A., Filho P.B., Dos Santos G.B., Przybyla G. (2020). National Potential Production of Methane and Electrical Energy from Sugarcane Vinasse in Brazil: A Thermo-Economic Analysis. J. Environ. Chem. Eng..

[2] Freitas P.V., Da Silva D.R., Beluomini M.A., Da Silva J.L., Stradiotto N.R. (2018). Determination of Phenolic Acids in Sugarcane Vinasse by HPLC with Pulse Amperometry. J. Anal. Methods Chem..

[3] Muñoz-Palazon B., Rodriguez-Sanchez A., Hurtado-Martinez M., de Castro I.M., Juarez-Jimenez B., Gonzalez-Martinez A., Gonzalez-Lopez J. (2019). Performance and Microbial Community Structure of an Aerobic Granular Sludge System at Different Phenolic Acid Concentrations. J. Hazard. Mater..

[4] Moreira V.R., Carpanez T.G., dos Santos F.S., Santos L.S., dos Santos Fernandes D., França-Neta L.S., Lange L.C., Amaral M.C.S. (2022). Circular Economy in Biorefineries: Scale-up of Anaerobic/Aerobic Membrane Bioreactors for Vinasse Recycling. J. Clean. Prod..

[5] Montiel-Rosales A., Montalvo-Romero N., García-Santamaría L.E., Sandoval-Herazo L.C., Bautista-Santos H., Fernández-Lambert G. (2022). Post-Industrial Use of Sugarcane Ethanol Vinasse: A Systematic Review. Sustainability.

[6] Russo T., Fucile P., Giacometti R., Sannino F. (2021). Sustainable Removal of Contaminants by Biopolymers: A Novel Approach for Wastewater Treatment. Current State and Future Perspectives. Processes.

[7] Hubbe M.A., Beck K.R., O’Neal W.G., Sharma Y.C. (2012). Cellulosic Substrates for Removal of Pollutants from Aqueous Systems: A Review. 2. Dyes. BioResources.

[8] Martínez-Ruiz Y., Manotas-Duque D.F., Osorio-Gómez J.C., Ramírez-Malule H. (2022). Evaluation of Energy Potential from Coffee Pulp in a Hydrothermal Power Market through System Dynamics: The Case of Colombia. Sustainability.

[9] Cerino-Córdova F.J., Dávila-Guzmán N.E., García León A.M., Salazar-Rabago J.J., Soto-Regalado E., Toledo Castanheira D. (2020). Revalorization of Coffee Waste. Coffee—Production and Research.

[10] Din M.F.M., Ponraj M., Low W.-P., Fulazzaky M.A., Iwao K., Songip A.R., Chelliapan S., Ismail Z., Jamal M.H. (2016). Removal Rate of Organic Matter Using Natural Cellulose via Adsorption Isotherm and Kinetic Studies. Water Environ. Res..

[11] Başaran Kankılıç G., Metin A.Ü. (2020). Phragmites Australis as a New Cellulose Source: Extraction, Characterization and Adsorption of Methylene Blue. J. Mol. Liq..

[12] Lapina V.A., Akhremkova G.S. (2006). Correlations between the Adsorption and Structural Properties of SV-1 Phytoadsorbent and Its Main Components. Russ. J. Phys. Chem. A.

[13] Ali Z.M., Mughal M.A., Laghari A.J., Ansari A.K., Saleem H. (2013). Polymeric Cellulose Derivative: Carboxymethyl-Cellulose as Useful Organic Flocculant against Industrial Waste Waters. Int. J. Adv. Res. Technol..

[14] Laverde-Rojas V., Liscano Y., Rivera-Sánchez S.P., Ocampo-Ibáñez I.D., Betancourt Y., Alhajj M.J., Yarce C.J., Salamanca C.H., Oñate-Garzón J. (2020). Antimicrobial Contribution of Chitosan Surface-Modified Nanoliposomes Combined with Colistin against Sensitive and Colistin-Resistant Clinical Pseudomonas Aeruginosa. Pharmaceutics.

[15] Bakshi P.S., Selvakumar D., Kadirvelu K., Kumar N.S. (2020). Chitosan as an Environment Friendly Biomaterial—A Review on Recent Modifications and Applications. Int. J. Biol. Macromol..

[16] González-Delgado Á.D., Moreno-Sader K.A., Martínez-Consuegra J.D. (2022). Biorrefinación Sostenible Del Camarón: Desarrollos Desde La Ingeniería de Procesos Asistida Por Computador.

[17] Sánchez Orozco M.D., Angarita Peñaranda M.R., Ortiz Ortega D.A., Rosas L.A. (2018). Harina de Subproductos de Camarón Como Oportunidad de Inclusión en Dietas para Alimentación Animal. RAA.

[18] Wang J.P., Chen Y.Z., Yuan S.J., Sheng G.P., Yu H.Q. (2009). Synthesis and Characterization of a Novel Cationic Chitosan-Based Flocculant with a High Water-Solubility for Pulp Mill Wastewater Treatment. Water Res..

[19] Poole C.F., Gunatilleka A.D., Sethuraman R. (2000). Contributions of Theory to Method Development in Solid-Phase Extraction. J. Chromatogr. A.

[20] Simpson N.J.K. (2000). Solid-Phase Extraction: Principles, Techniques, and Applications.

[21] Runge M.B., Bowden N.B. (2007). Synthesis of High Molecular Weight Comb Block Copolymers and Their Assembly into Ordered Morphologies in the Solid State. J. Am. Chem. Soc..

[22] De Alvarenga G., Hryniewicz B.M., Jasper I., Silva R.J., Klobukoski V., Costa F.S., Cervantes T.N.M., Amaral C.D.B., Schneider J.T., Bach-Toledo L. (2020). Recent Trends of Micro and Nanostructured Conducting Polymers in Health and Environmental Applications. J. Electroanal. Chem..

[23] Ali I., Gupta V.K. (2006). Advances in Water Treatment by Adsorption Technology. Nat. Protoc..

[24] Reyes I., Palacio M.M., Yarce C.J., Oñate-Garzón J., Salamanca C.H. (2020). Relationship between the Ionization Degree and the Inter-Polymeric Aggregation of the Poly(Maleic Acid-Alt-Octadecene) Salts Regarding Time. Polymers.

[25] Subash A., Naebe M., Wang X., Kandasubramanian B. (2023). Biopolymer—A Sustainable and Efficacious Material System for Effluent Removal. J. Hazard. Mater..

[26] Nairat N., Hamed O., Berisha A., Jodeh S., Algarra M., Azzaoui K., Dagdag O., Samhan S. (2022). Cellulose Polymers with β-Amino Ester Pendant Group: Design, Synthesis, Molecular Docking and Application in Adsorption of Toxic Metals from Wastewater. BMC Chem..

[27] Khalaf B., Hamed O., Jodeh S., Bol R., Hanbali G., Safi Z., Dagdag O., Berisha A., Samhan S. (2021). Cellulose-Based Hectocycle Nanopolymers: Synthesis, Molecular Docking and Adsorption of Difenoconazole from Aqueous Medium. Int. J. Mol. Sci..

[28] Park S.-J., Kern N., Brown T., Lee J., Im W. (2023). CHARMM-GUI PDB Manipulator: Various PDB Structural Modifications for Biomolecular Modeling and Simulation. J. Mol. Biol..

[29] Jo S., Kim T., Iyer V.G., Im W. (2008). CHARMM-GUI: A Web-Based Graphical User Interface for CHARMM. J. Comput. Chem..

[30] Abraham M.J., Murtola T., Schulz R., Páll S., Smith J.C., Hess B., Lindahl E. (2015). GROMACS: High Performance Molecular Simulations through Multi-Level Parallelism from Laptops to Supercomputers. SoftwareX.

[31] Grubmüller H., Heller H., Windemuth A., Schulten K. (1991). Generalized Verlet Algorithm for Efficient Molecular Dynamics Simulations with Long-Range Interactions. Mol. Simul..

[32] Harvey M.J., De Fabritiis G. (2009). An Implementation of the Smooth Particle Mesh Ewald Method on GPU Hardware. J. Chem. Theory Comput..

[33] Van Der Spoel D., Lindahl E., Hess B., Groenhof G., Mark A.E., Berendsen H.J.C. (2005). GROMACS: Fast, Flexible, and Free. J. Comput. Chem..

[34] Nielsen S.O. (2013). Nested Sampling in the Canonical Ensemble: Direct Calculation of the Partition Function from NVT Trajectories. J. Chem. Phys..

[35] Bernetti M., Bussi G. (2020). Pressure Control Using Stochastic Cell Rescaling. J. Chem. Phys..

[36] Sharma S. (2019). Molecular Dynamics Simulation of Nanocomposites Using BIOVIA Materials Studio, Lammps and Gromacs.

[37] Kumari R., Kumar R. (2014). Open Source Drug Discovery Consortium; Lynn, A. G_mmpbsa —A GROMACS Tool for High-Throughput MM-PBSA Calculations. J. Chem. Inf. Model..

[38] Baker N.A., Sept D., Joseph S., Holst M.J., McCammon J.A. (2001). Electrostatics of Nanosystems: Application to Microtubules and the Ribosome. Proc. Natl. Acad. Sci. USA.

[39] Guo Z., Lue B.-M., Thomasen K., Meyer A.S., Xu X. (2007). Predictions of Flavonoid Solubility in Ionic Liquids by COSMO-RS: Experimental Verification, Structural Elucidation, and Solvation Characterization. Green Chem..

[40] Alara O.R., Abdurahman N.H., Ukaegbu C.I. (2021). Extraction of Phenolic Compounds: A Review. Curr. Res. Food Sci..

[41] Liu Y., Ying D., Sanguansri L., Cai Y., Le X. (2018). Adsorption of Catechin onto Cellulose and Its Mechanism Study: Kinetic Models, Characterization and Molecular Simulation. Food Res. Int..

[42] Behra J.S., Mattsson J., Cayre O.J., Robles E.S.J., Tang H., Hunter T.N. (2019). Characterization of Sodium Carboxymethyl Cellulose Aqueous Solutions to Support Complex Product Formulation: A Rheology and Light Scattering Study. ACS Appl. Polym. Mater..

[43] Capanema N.S.V., Mansur A.A.P., Mansur H.S., De Jesus A.C., Carvalho S.M., Chagas P., De Oliveira L.C. (2018). Eco-Friendly and Biocompatible Cross-Linked Carboxymethylcellulose Hydrogels as Adsorbents for the Removal of Organic Dye Pollutants for Environmental Applications. Environ. Technol..

[44] Liudvinaviciute D., Almonaityte K., Rutkaite R., Bendoraitiene J., Klimaviciute R. (2018). Adsorption of Rosmarinic Acid from Aqueous Solution on Chitosan Powder. Int. J. Biol. Macromol..

[45] Flores-Chaparro C.E., Rodriguez-Hernandez M.C., Chazaro-Ruiz L.F., Alfaro-De La Torre M.C., Huerta-Diaz M.A., Rangel-Mendez J.R. (2018). Chitosan-Macroalgae Biocomposites as Potential Adsorbents of Water-Soluble Hydrocarbons: Organic Matter and Ionic Strength Effects. J. Clean. Prod..

[46] Kolář M., Kubař T., Hobza P. (2011). On the Role of London Dispersion Forces in Biomolecular Structure Determination. J. Phys. Chem. B.

[47] Meyer E., Rosenberg K.J., Israelachvili J. (2006). Recent progress in understanding hydrophobic interactions. Proc. Natl. Acad. Sci. USA.

